# Type I IFN Induces IL-10 Production in an IL-27–Independent Manner and Blocks Responsiveness to IFN-γ for Production of IL-12 and Bacterial Killing in *Mycobacterium tuberculosis*–Infected Macrophages

**DOI:** 10.4049/jimmunol.1401088

**Published:** 2014-09-03

**Authors:** Finlay W. McNab, John Ewbank, Ashleigh Howes, Lucia Moreira-Teixeira, Anna Martirosyan, Nico Ghilardi, Margarida Saraiva, Anne O’Garra

**Affiliations:** * Division of Immunoregulation, Medical Research Council National Institute for Medical Research, London NW7 1AA, United Kingdom;; † Life and Health Sciences Research Institute, School of Health Sciences, University of Minho, 4710-057 Braga, Portugal;; ‡ Life and Health Sciences Research Institute and Biomaterials, Biodegradables and Biomimetics Research Group, Portugal Government Associate Laboratory, 4710-057 Braga/Guimarães, Portugal; and; § Department of Immunology, Genentech, Inc., South San Francisco, CA 94080

## Abstract

Tuberculosis, caused by the intracellular bacterium *Mycobacterium tuberculosis*, currently causes ∼1.4 million deaths per year, and it therefore remains a leading global health problem. The immune response during tuberculosis remains incompletely understood, particularly regarding immune factors that are harmful rather than protective to the host. Overproduction of the type I IFN family of cytokines is associated with exacerbated tuberculosis in both mouse models and in humans, although the mechanisms by which type I IFN promotes disease are not well understood. We have investigated the effect of type I IFN on *M. tuberculosis*–infected macrophages and found that production of host-protective cytokines such as TNF-α, IL-12, and IL-1β is inhibited by exogenous type I IFN, whereas production of immunosuppressive IL-10 is promoted in an IL-27–independent manner. Furthermore, much of the ability of type I IFN to inhibit cytokine production was mediated by IL-10. Additionally, type I IFN compromised macrophage activation by the lymphoid immune response through severely disrupting responsiveness to IFN-γ, including *M. tuberculosis* killing. These findings describe important mechanisms by which type I IFN inhibits the immune response during tuberculosis.

## Introduction

Myeloid cells such as macrophages are the predominant targets of *Mycobacterium tuberculosis* infection (1–3). Effector functions elicited in these cells upon infection are crucial to the establishment of an adaptive immune response to *M. tuberculosis*, restriction of bacterial growth, and ultimately to host resistance (1–3). Key effectors linked to host protection produced by these cells include the cytokines IL-12, TNF-α, and IL-1α/β (1–3). Production of NO and other molecules that restrict intracellular bacterial growth is also of importance (4–6). IL-12 is critical for activation of CD4^+^ T cells, leading to the protective Th1 response and induction of IFN-γ from a variety of cellular sources (7, 8). IFN-γ is in turn crucial for the full activation of macrophage bactericidal functions (such as NO production and restriction of mycobacterial growth) and further enhancement of innate cytokine production (5, 6, 9–14).

Whereas immune mechanisms that lead to host resistance have been intensively studied, less is understood about potentially damaging or inhibitory immune responses leading to activation or exacerbation of tuberculosis (TB). There is a growing appreciation that the type I IFN family of cytokines plays a detrimental as opposed to protective role during TB, particularly when such cytokines are present in excess amounts, with evidence in both mice and humans largely supporting this hypothesis (15–25). Studies of *M. tuberculosis* infection of mice that lack the receptor common to all type I IFN (*Ifnar1*^−/−^ mice) found reduced bacterial load and/or increased survival compared with wild-type (WT) mice (18, 19, 22, 24), pointing to a negative role of type I IFN in TB. However, results have not been unequivocal, with some suggestion that type I IFN may have protective activities under certain conditions (26, 27).

Studies where excess levels of type I IFN are induced during TB, either through direct instillation of IFN-α/β into the lung (18), administration of a polyinosinic-polycytidylic acid derivative (15), or abrogation of a negative regulator of type I IFN signaling (28) support a detrimental role for type I IFN during TB, as they all resulted in exacerbated disease. In accordance with these results, hypervirulent *M. tuberculosis* strains reportedly induce high levels of type I IFN (18, 19).

Recent reports have also revealed a potential role for type I IFN in the human immune response during TB, because cohorts of active TB patients in London and South Africa showed a prominent type I IFN–inducible gene signature in their blood, which cell separation experiments found was present predominantly in myeloid cells (16). Furthermore, the signature was correlated with the extent of radiographic disease and resolved upon successful treatment (16). These findings have now been verified by other groups using patient cohorts from Africa (17, 29) and Indonesia (23).

The mechanisms by which type I IFN exacerbates disease during *M. tuberculosis* infection are only partially understood. Recently, studies have described a role for type I IFN in suppressing production of the protective cytokine IL-1 in both in vivo mouse models (20) and human monocytes (21). This inhibition of IL-1 was partially dependent on IL-10 (20), which is known to be induced by type I IFN (30–32). IL-10 is also induced during infection with another intracellular bacteria, *Listeria monocytogenes*, where type I IFNs are similarly detrimental to the host (33–36). Furthermore, IL-10 is known to inhibit the immune response during TB, particularly the Th1 cell response (37–41). Earlier studies of infection with hypervirulent *M. tuberculosis* strains that induce high levels of type I IFN also point to a reduction in the protective Th1 response, with reduced IL-12 and IFN-γ during infection (18). In human infections with the leprosy agent *Mycobacterium leprae*, type I IFN has also been shown to induce IL-10 and restrict IFN-γ–mediated antibacterial responses (42). Additionally, type I IFNs have been shown to downregulate IFN-γ receptor expression on myeloid cells during *L. monocytogenes* infection (43).

We have investigated the effects of type I IFN on macrophage function during *M. tuberculosis* infection. We found that type I IFN inhibits production of multiple protective cytokines by *M. tuberculosis*–infected macrophages and induces the immunosuppressive cytokine IL-10. Notably, type I IFN also robustly suppressed macrophage responsiveness to IFN-γ, preventing IFN-γ–dependent enhancement of cytokine production and inhibiting IFN-γ–mediated macrophage bacterial growth inhibition and killing. These results suggest an important role for type I IFN in inhibiting the immune response to *M. tuberculosis* at the level of the macrophage, potentially impairing both macrophage induction of, and response to, adaptive immunity.

## Materials and Methods

### Mice

C57BL/6 (B6), B6 *Ifnar1*^−/−^, B6 *Il10*^−/−^, and B6 *Tccr*^−/−^ (IL-27R α-chain–deficient) mice were bred and housed under specific pathogen-free conditions at the Medical Research Council National Institute for Medical Research, B6 *Nos2*^−/−^ mice (and B6 WT controls) were bred and housed under specific pathogen-free conditions at the Instituto de Biologia Molecular e Celular (Porto, Portugal), and bones from these mice were shipped overnight on ice to the National Institute for Medical Research. All protocols for breeding and experiments were performed in accordance with either Home Office (U.K.) requirements and the Animal Scientific Procedures Act, 1986 or according to the European Union directive 86/609/EEC and approved by the Portugese national authority for animal health, Direcção Geral de Veterinária (Portugal). Mice were sex and age matched for use in experiments.

### Reagents

Cell culture medium was RPMI 1640 (Lonza) supplemented with 5% heat-inactivated FCS (Biosera), 0.05 mM 2-ME (Sigma-Aldrich), 2 mM l-glutamine (Lonza), 1 mM sodium pyruvate (Lonza), and 10 mM HEPES (Lonza). rIFN-β was purchased from PBL Assay Science and rIFN-γ was purchased from R&D Systems. IFN-β was used at 2 ng/ml unless otherwise indicated, and IFN-γ was used at 5 ng/ml. Anti–IL-10R (clone 1B1.3A) and anti–IL-10 (clone TC40.11D8) mAbs and their isotype controls (GL113 and TC31.2F11, respectively) were gifts from DNAX Research Institute (now Merck, Palo Alto, CA) and were used at 10 μg/ml.

### Generation and infection of murine bone marrow–derived macrophages and enrichment and infection of bone marrow and lung myeloid cells

Bone marrow (BM) cells were flushed from the femurs and tibias of mice and plated at 0.5 × 10^6^ cells/ml on bacterial plates (Sterilin) in culture medium containing 10% FCS and 20% L929 cell–conditioned medium. At day 6, macrophages were harvested and seeded into 24-well tissue culture plates (Corning) at 1 × 10^6^ cells/ml. Cells were rested overnight, washed once with PBS, and infected at a multiplicity of infection of 2:1 with *M. tuberculosis* H37Rv and treated where indicated with recombinant cytokines and/or mAbs. All experiments using *M. tuberculosis* were carried out under biosafety containment level 3 conditions. *M. tuberculosis* H37Rv was grown as previously described (39). *M. tuberculosis* was left in the wells until the supernatant or cells were harvested, unless otherwise stated. The number of bacteria in the inoculum was determined by serial dilutions on 7H11 plates supplemented with 10% OADC. For mRNA stability experiments cells were treated at 1 h after *M. tuberculosis* infection with 10 μg/ml actinomycin D (ActD) and then harvested for RNA at indicated time points after ActD treatment.

BM and lung myeloid cells were enriched from whole-organ cell suspensions using EasySep (StemCell Technologies) magnetic separation protocols. Cell suspensions were first enriched using the mouse monocyte enrichment kit (StemCell Technologies, catalog no. 19761A), followed by staining with anti-Ly6c FITC Ab (BD Pharmingen, clone AL-21) and further enrichment with the mouse FITC selection kit (StemCell Technologies, catalog no. 18515). Cells were then plated at 0.5 × 10^6^ cells/ml and infected with H37Rv at a multiplicity of infection of 2:1 and treated where indicated with rIFN-β and/or rIFN-γ.

### Enumeration of intracellular *M. tuberculosis* in macrophages following infection

To determine the number of intracellular *M. tuberculosis* CFUs present in macrophages following infection, supernatants were harvested, macrophages were washed once with PBS to remove extracellular bacteria, and 1 ml 0.2% saponin (Sigma-Aldrich) was added for 1 h at 37°C to lyse the cells. This suspension was then serially diluted and plated onto 7H11 plates supplemented with OADC, and colonies were counted after 14–16 d at 37°C.

### Cytokine quantification by ELISA and bead array

Cytokine concentrations in the supernatants of infected cells were determined by ELISA or Luminex bead array at 24 h after *M. tuberculosis* infection. This time point was chosen based on pilot experiments to determine the optimal postinfection time point for analysis. Commercially available kits were used for TNF-α, IL-12p70, IL-27 (all eBioscience), and IL-1β (R&D Systems) and were used according to the manufacturers’ instructions. Matched Ab pairs were used for IL-12p40 and IL-10. IL-12p40 was detected using Ab clone C15.6.7 for capture and biotinylated Ab clone C17.8 for detection. IL-10 was detected using Ab clone JES5-2A5 for capture and biotinylated anti–IL-10 for detection (BD Biosciences, clone SXC-1). Custom magnetic bead arrays to measure cytokines in supernatant of primary ex vivo cells were purchased from Millipore (Merck Millipore, Billerica, MA) and used according to the manufacturer’s instructions. Samples were run on a Bio-Rad Luminex 200 machine (Bio-Rad, Hercules, CA). Cytokine levels from uninfected cells were below the assay level of detection unless otherwise shown (20 pg/ml for ELISA, 5 pg/ml for bead array) (data not shown).

### Processing of macrophage RNA and quantitative PCR analysis

At indicated times postinfection, supernatants were removed and cells were washed once with PBS. RNA was harvested in 350 μl RLT buffer (Qiagen) and stored at −80°C before processing. RNA was processed using RNeasy Mini kits (Qiagen). RNA was reverse transcribed with a high-capacity reverse transcription kit (Applied Biosystems) to cDNA. The expression of indicated genes was quantified by real-time PCR (ABI Prism 7900 from Applied Biosystems) and normalized against *Hprt* mRNA levels. Murine primers were all purchased from Applied Biosystems.

### Protein analysis and Western blotting

Spleens were homogenized by passing through a 70-μm sieve, and cell suspensions were then RBC lysed and cultured in media containing 1% FCS for 5 h before treatment with rIL-27 (50 ng/ml), rIFN-γ (10 ng/ml), or rIL-10 (10 ng/ml) for the indicated times. Where indicated cells were treated with 200 ng/ml Pam3CSK4 (Invivogen). BM-derived macrophages (BMDMs) were grown as described above but rested overnight in 1% FCS prior to treatment with recombinant cytokines and stimuli, as indicated. Cells were then harvested, lysed in RIPA buffer, and immunoblotting was carried out as previously described (44). Anti–phospho-STAT1 (Y701), anti–total STAT1, anti–phospho-STAT3 (Y705), anti–total STAT3 (all Cell Signaling Technology), and anti-actin (Calbiochem) primary Abs, followed by HRP-conjugated goat anti-rabbit IgG (SouthernBiotech) or goat anti-mouse IgM (Calbiochem) secondary Abs, were used to probe membranes.

### Statistical analysis

Statistical analysis was carried out using Prism software version 6 (GraphPad Software). Statistical tests used to determine significance are described in the figure legends with values as follows: *p < 0.05, **p < 0.01, and ***p < 0.001.

## Results

### Type I IFN regulates IL-10 production in *M. tuberculosis*–infected macrophages

**FIGURE 1.**
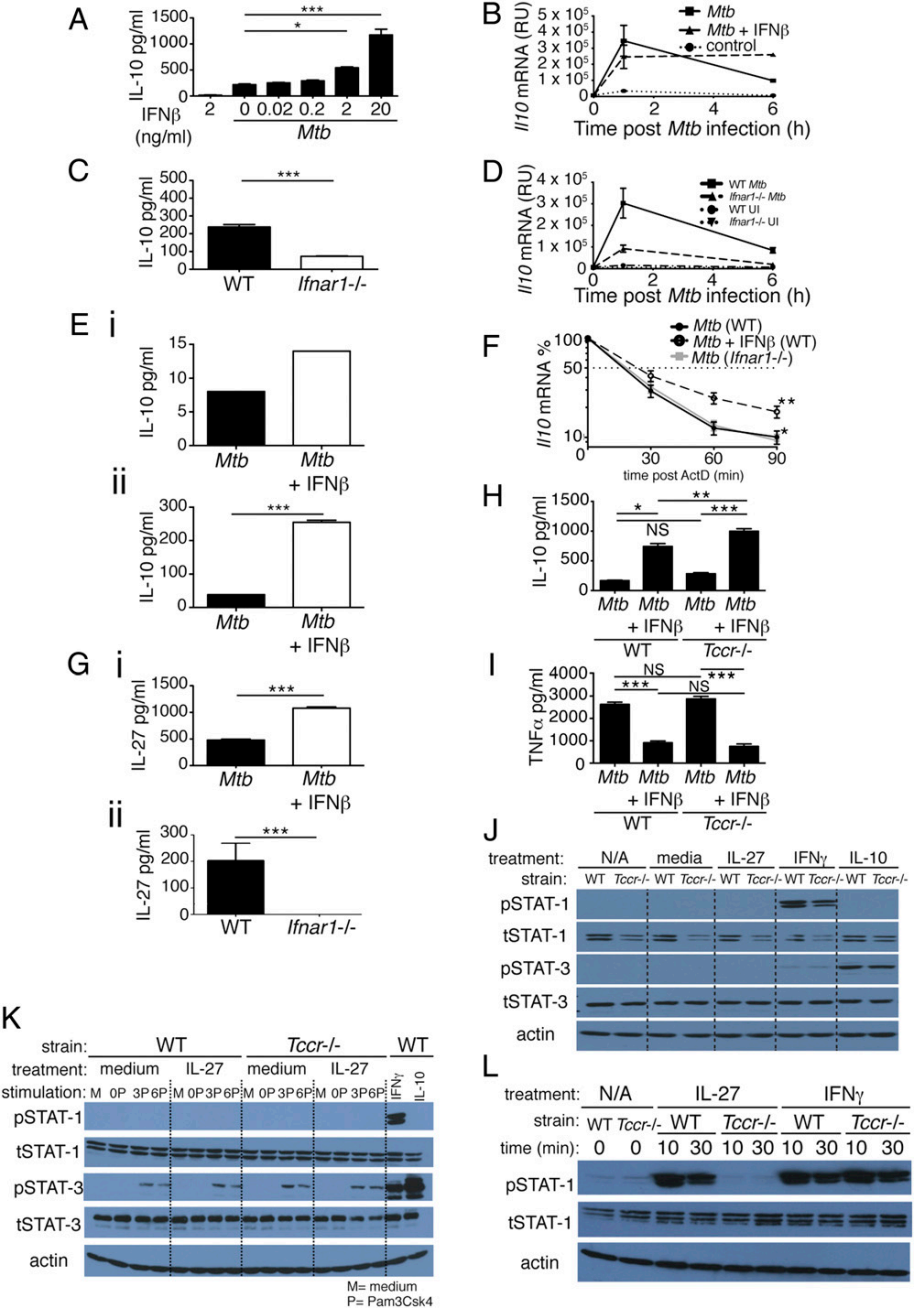
Type I IFN regulates IL-10 production in *M. tuberculosis*–infected macrophages independently of IL-27 signaling. (**A**) WT macrophages were infected with *M. tuberculosis* in the presence of increasing concentrations of IFN-β, added at the time of infection, and levels of IL-10 in culture supernatant were determined by ELISA at 24 h postinfection. (**B**) WT macrophages were infected with *M. tuberculosis* in the presence or absence of 2 ng/ml IFN-β, added at the time of infection, and levels of *Il10* mRNA were determined by quantitative RT-PCR (qRT-PCR) at the time points indicated after infection. (**C**) WT and *Ifnar1*^−/−^ macrophages were infected with *M. tuberculosis* and levels of IL-10 in culture supernatant were determined by ELISA at 24 h postinfection. (**D**) WT and *Ifnar1*^−/−^ macrophages were infected with *M. tuberculosis* and levels of *Il10* mRNA determined by qRT-PCR at the time points indicated after infection. (**E**) WT myeloid cells taken ex vivo from lungs (**i**) and BM (**ii**) were infected with *M. tuberculosis* in the presence or absence of 2 ng/ml IFN-β, added at the time of infection, and levels of IL-10 in culture supernatant were determined by Luminex bead array at 24 h postinfection. (**F**) WT, *Ifnar1*^−/−^, and WT treated with IFN-β macrophages were infected with *M. tuberculosis*, and at 1 h postinfection ActD was added. mRNA was then taken at the time points indicated and *Il10* mRNA levels were determined by qRT-PCR. (**G**) WT macrophages treated (or not) with 2 ng/ml IFN-β at the time of infection (**i**) and WT and *Ifnar1*^−/−^ macrophages (**ii**) were infected with *M. tuberculosis*, and levels of IL-27 in culture supernatant were determined by ELISA at 24 h postinfection. (**H** and **I**) WT and *Tccr*^−/−^ (IL-27Rα^−/−^) macrophages were infected with *M. tuberculosis* in the presence or absence of 2 ng/ml IFN-β, added at the time of infection, and levels of IL-10 (H) or TNF-α (I) in culture supernatant were determined by ELISA at 24 h postinfection. (**J**) WT and *Tccr*^−/−^ (IL-27Rα^−/−^) macrophages were treated for 20 min with rIL-27 (50 ng/ml), rIFN-γ (10 ng/ml), or rIL-10 (10 ng/ml) and whole-cell extracts were then analyzed by immunoblotting with the indicated Abs. (**K**) WT and *Tccr*^−/−^ (IL-27Rα^−/−^) macrophages were stimulated for 0, 3, or 6 h with Pam3CSK4 (200 ng/ml) and then treated (or not) for 20 min with rIL-27 (50 ng/ml). WT macrophages treated with IFN-γ (10 ng/ml) or IL-10 (10 ng/ml) were included as positive controls for STAT-1 and STAT-3 phosphorylation, respectively. Whole-cell extracts were then analyzed by immunoblotting with the indicated Abs. (**L**) WT and *Tccr*^−/−^ splenocytes were treated for the indicated times with rIL-27 (50 ng/ml) or rIFN-γ (10 ng/ml). Whole-cell extracts were then analyzed by immunoblotting with the indicated Abs. Graphs show means ± SEM of triplicate samples, except for (E), which shows duplicates. For ELISA and Luminex bead array results, uninfected control samples were below the detection limit (20 and 5 pg/ml, respectively) for the cytokines measured (data not shown). Significance was determined using an unpaired *t* test (A, C, E, and G), a one-way ANOVA with a Bonferroni post hoc test (H and I), or a two-way ANOVA, with significance relative to WT (F). Data are representative of at least two independent experiments. *p < 0.05, **p < 0.01, ***p < 0.001.

Although type I IFN has been implicated in exacerbation of TB, it remains unclear how it manifests its effects at the molecular level. We investigated whether type I IFN regulated production of IL-10, an immunosuppressive cytokine generally linked to TB exacerbation (37–41). Initially, we assessed whether type I IFN could enhance IL-10 production by infecting WT macrophages with *M. tuberculosis* and concomitantly adding rIFN-β at varying concentrations from 0.02 to 20 ng/ml (Fig. 1A). Addition of IFN-β significantly enhanced IL-10 production by *M. tuberculosis*–infected macrophages when added at 2 and 20 ng/ml (Fig. 1A). Notably, type I IFN on its own was not sufficient to induce IL-10 production by macrophages, in support of a role for this cytokine as an enhancer of IL-10 production (Fig. 1A). Additionally, we assessed the effects of IFN-β addition on *Il10* mRNA transcription (Fig. 1B). Addition of 2 ng/ml IFN-β to *M. tuberculosis*–infected macrophages resulted in enhanced *Il10* mRNA levels at 6 h postinfection (Fig. 1B).

We also investigated the effect of autocrine type I IFN on macrophage IL-10 production in response to *M. tuberculosis* by infecting WT and *Ifnar1*^−/−^ macrophages with *M. tuberculosis* H37Rv (Fig. 1C). *Ifnar1*^−/−^ macrophages produced significantly less IL-10 protein compared with WT macrophages following *M. tuberculosis* infection (Fig. 1C), and *Il10* mRNA transcription was greatly reduced at 1 and 6 h postinfection in the *Ifnar1*^−/−^ macrophages (Fig. 1D). We then examined myeloid cells taken directly ex vivo from lungs and BM of mice for their ability to produce IL-10 following *M. tuberculosis* infection and IFN-β treatment (Fig. 1E). As with BMDMs, IFN-β increased the levels of IL-10 produced by *M. tuberculosis*–infected ex vivo myeloid cells from lung (Fig. 1Ei) and BM (Fig. 1Eii). Thus, our data support a role for type I IFN signaling in inducing IL-10 production by *M. tuberculosis*–infected macrophages, likely through transcriptional regulation. Addition of IFN-β to *M. tuberculosis*–infected macrophages led to stable *Il10* mRNA between 1 and 6 h (Fig. 1B), suggestive of some effect on mRNA stability. To investigate this further we performed experiments to assess *Il10* mRNA stability in the presence or absence of type I IFN signaling (Fig. 1F). WT macrophages, with or without addition of rIFN-β, and *Ifnar1*^−/−^ macrophages were infected with *M. tuberculosis* and 1 h after infection ActD was added to the cultures to inhibit further transcription. *Il10* mRNA was then measured at 0, 30, 60, and 90 min after ActD addition and plotted as a percentage of the *Il10* mRNA level in the 0 min ActD-treated samples (Fig. 1F). Loss of type I IFN signaling did not greatly affect the decay of *Il10* mRNA levels over time, as WT and *Ifnar1*^−/−^ macrophages had a similar percentage of *Il10* mRNA over time after ActD treatment (Fig. 1F). However, addition of rIFN-β to infected WT macrophages increased *Il10* mRNA stability, as the percentage of *Il10* mRNA remaining in IFN-β–treated macrophages was significantly increased compared with *M. tuberculosis*–infected WT macrophages alone and *Ifnar1*^−/−^ macrophages (Fig. 1F). Thus, type I IFN increases *Il10* mRNA stability but is not absolutely required for *Il10* mRNA stability. Taken together, these data suggest that type I IFN likely regulates IL-10 levels through a combination of transcriptional control and modulation of *Il10* mRNA stability.

### IL-10 production, as well as enhancement of IL-10 production by type I IFN, is independent of IL-27 signaling in *M. tuberculosis*–infected macrophages

IL-27 is known to induce IL-10 production by T cells (45–47) and has been suggested to act as an intermediate between LPS-induced type I IFN and IL-10 production by macrophages (32). However, in human monocytes (48) and some dendritic cells (49) it has been reported that IL-27 inhibits IL-10 production. Furthermore, it has been suggested that murine BMDMs are minimally responsive to IL-27 (48). We therefore investigated a role for IL-27 in IL-10 induction by type I IFN during *M. tuberculosis* infection of macrophages. Initially we determined whether type I IFN was required for IL-27 production by *M. tuberculosis*–infected macrophages. Addition of IFN-β to WT macrophages upon *M. tuberculosis* infection led to enhanced IL-27 production (Fig. 1Gi), in agreement with previous studies with LPS-stimulated macrophages (32). Furthermore, infection of WT and *Ifnar1*^−/−^ macrophages revealed that type I IFN signaling was required for IL-27 production upon *M. tuberculosis* infection (Fig. 1Gii).

We then investigated whether IL-27 was required for IL-10 production in *M. tuberculosis*–infected macrophages, and also for promotion of IL-10 production by type I IFN. This was done by infecting WT and *Tccr*^−/−^ macrophages (that lack the IL-27R α-chain and hence a functional IL-27 receptor) (50) with *M. tuberculosis* in the presence or absence of IFN-β (Fig. 1H). As expected, *M. tuberculosis*–infected WT macrophages produced IL-10 and the levels of IL-10 were increased by addition of IFN-β (Fig. 1H). *Tccr*^−/−^ macrophages also produced IL-10 in response to *M. tuberculosis* infection, and levels were also increased upon addition of IFN-β (Fig. 1H). Levels of TNF-α and other proinflammatory cytokines were not reproducibly different between WT and *Tccr*^−/−^ macrophages infected with *M. tuberculosis* (Fig. 1I and data not shown). Resting BMDMs expressed only low levels of the transcripts for both IL-27R subunits compared with naive T cells (Supplemental Fig. 1A). LPS- or CpG-treated BMDMs (Supplemental Fig. 1B), BM-derived dendritic cells, and BM monocytes (data not shown) did not produce different levels of IL-10 following treatment with rIL-27. These data suggest that IL-27R signaling is not required for IL-10 production by *M. tuberculosis*–infected macrophages or for IFN-β–mediated enhancement of IL-10 production by *M. tuberculosis*–infected macrophages.

It has previously been suggested that IL-27 induces IL-10 downstream of type I IFN in BMDMs in a STAT-1– and STAT-3–dependent manner (32). To investigate whether WT BMDMs activated STAT-1 and STAT-3 following IL-27 treatment and to confirm that *Tccr*^−/−^ cells did not transduce any signal from IL-27, we treated BMDMs from WT and *Tccr*^−/−^ mice with rIL-27 for 20 min and then probed by Western blot for phosphorylation of STAT-1 and STAT-3 (Fig. 1J). Neither STAT-1 nor STAT-3 was phosphorylated in response to IL-27 in both WT and *Tccr*^−/−^ macrophages (Fig. 1J). BMDMs of both genotypes were capable of phosphorylating STAT-1 and STAT-3, because p–STAT-1 and p–STAT-3 were detected in both in response to IFN-γ or IL-10 addition, which led to phosphorylation of STAT-1 and STAT-3, respectively (Fig. 1J).

To control for the possibility that WT BMDMs might upregulate IL-27R following TLR stimulation and thereby activate STAT-1 and/or STAT-3 in response to IL-27 treatment in the presence of TLR activation, we stimulated WT and *Tccr*^−/−^ macrophages with the TLR2 agonist Pam3CSK4 for 0, 3, or 6 h, treated them with IL-27 for 20 min, and then assayed for phosphorylation of STAT-1 and STAT-3 using Western blot (Fig. 1K). We specifically chose Pam3CSK4 as a stimulus, as it does not activate STAT-1 and only activates low levels of STAT-3 (data not shown) and hence would minimally confound any effects of IL-27 that other stimuli that activate these STATs would. STAT-3 was phosphorylated in both WT and *Tccr*^−/−^ macrophages 3 and 6 h after stimulation with Pam3CSK4, but this was not enhanced further by treatment with IL-27 or reduced in *Tccr*^−/−^ cells (Fig. 1K). STAT-1 phosphorylation was not induced under these conditions but could be induced by addition of IFN-γ (Fig. 1K).

However, rIL-27 was capable of activating signaling in other WT cells, because STAT-1 was phosphorylated in whole splenocytes treated with IL-27 for 10 or 30 min but not in *Tccr*^−/−^ splenocytes, demonstrating both the activity of the recombinant cytokine and the specificity of the knockout (Fig. 1L). These data confirm that the IL-27R in the *Tccr*^−/−^ mice used is nonfunctional.

Collectively, our data demonstrate that although IL-27 is induced by type I IFN, IL-27 is not required for type I IFN–mediated IL-10 upregulation by *M. tuberculosis*–infected macrophages.

### Exogenous IFN-β inhibits IL-12, TNF, and IL-1β production in *M. tuberculosis*–infected macrophages despite type I IFN signaling being required for optimal IL-12p40 and TNF-α production

**FIGURE 2.**
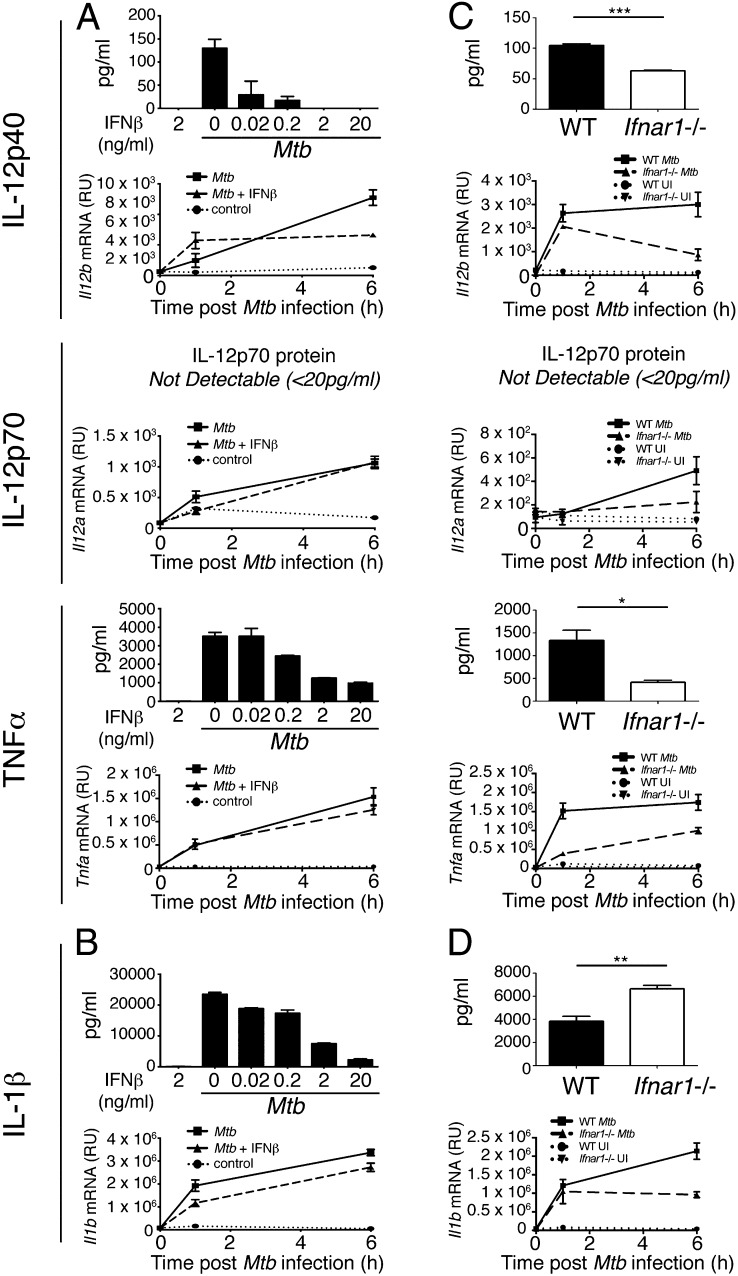
Opposing effects of exogenous IFN-β treatment and autocrine type I IFN signaling on IL-12 and TNF-α production in *M. tuberculosis*–infected macrophages. (**A**) WT macrophages were infected with *M. tuberculosis* in the presence of increasing concentrations of IFN-β (for protein) or 2 ng/ml IFN-β (for mRNA), added at the time of infection, and levels of IL-12p40, IL-12p70, and TNF-α protein in supernatant and *Il12b*, *Il12a*, and *Tnfa* mRNA from cells were determined by ELISA at 24 h postinfection and qRT-PCR at indicated times after infection. (**B**) IL-1β protein and *Il1b* mRNA from macrophages in (A) were measured by ELISA at 24 h postinfection and qRT-PCR at indicated times after infection. (**C**) WT and *Ifnar1*^−/−^ macrophages were infected with *M. tuberculosis* and IL-12p40, IL-12p70, and TNF-α protein in supernatant and *Il12b*, *Il12a*, and *Tnfa* mRNA from cells were determined by ELISA at 24 h postinfection and qRT-PCR at indicated times after infection. (**D**) IL-1β protein and *Il1b* mRNA from macrophages in (C) were measured by ELISA at 24 h postinfection and qRT-PCR at indicated times after infection. Graphs show means ± SEM of triplicate samples. For ELISA results, uninfected control samples were below the detection limit (20 pg/ml) for the cytokines measured (data not shown). Significance was determined using an unpaired *t* test. Data are representative of at least three independent experiments. *p < 0.05, **p < 0.01, ***p < 0.001.

We next wanted to examine how type I IFN might affect production by macrophages of proinflammatory cytokines important in host protection against *M. tuberculosis*. During in vivo *M. tuberculosis* infection a number of diverse cell types may produce type I IFN to influence macrophage function. We therefore initially sought to assess the effects of exogenous sources of type I IFN on proinflammatory cytokine production by *M. tuberculosis*–infected macrophages by infecting WT macrophages with *M. tuberculosis* and concomitantly adding rIFN-β at varying doses (Fig. 2A, 2B). Addition of IFN-β to *M. tuberculosis*–infected macrophages inhibited both IL-12p40 and TNF-α production (Fig. 2A). IL12p40 production appeared to be more sensitive than TNF-α to IFN-β treatment, being inhibited greatly at doses as low as 0.02 ng/ml, whereas TNF-α production was only inhibited at doses of 0.2 ng/ml or higher (Fig. 2A). IL-12p70 protein was not detected in any group (limit of detection [LOD], 20 pg/ml) (Fig. 2A). Levels of gene transcription for these cytokines were also assessed following addition of 2 ng/ml IFN-β to *M. tuberculosis*–infected macrophages. *Il12b* mRNA was not significantly affected at 1 h postinfection by addition of IFN-β but was greatly decreased in IFN-β–treated, *M. tuberculosis*–infected macrophages at 6 h compared with *M. tuberculosis*–infected alone macrophages (Fig. 2A). Similarly, *Tnfa* mRNA levels were lower at 6 h postinfection in IFN-β–treated macrophages but not at 1 h (Fig. 2A). Conversely, IFN-β treatment reduced *Il12a* mRNA in infected macrophages compared with *M. tuberculosis*–infected alone macrophages at 1 h but not at 6 h postinfection (Fig. 2A). Levels of IL-1β production were also assessed in *M. tuberculosis*–infected macrophages treated with IFN-β (Fig. 2B). Similar to the findings for IL-12p40 and TNF-α, IL-1β production was inhibited by IFN-β addition, with IL-1β protein levels being reduced at doses of 0.02 ng/ml or higher (Fig. 2B). *Il1b* mRNA levels were also reduced following IFN-β treatment of *M. tuberculosis*–infected macrophages, being reduced at 1 and 6 h postinfection compared with *M. tuberculosis*–infected alone macrophages (Fig. 2B).

We next examined what effects autocrine type I IFN signaling had on proinflammatory cytokine production upon infection of WT and *Ifnar1*^−/−^ macrophages with *M. tuberculosis*. Unexpectedly, given the previous results with addition of IFN-β, the amount of IL-12p40 secreted was reduced in *M. tuberculosis*–infected *Ifnar1*^−/−^ macrophages compared with WT cells, as were the levels of *Il12b* mRNA at 1 and 6 h postinfection (Fig. 2C). IL-12p70 was not detectable from either WT or *Ifnar1*^−/−^ macrophages infected with *M. tuberculosis* (LOD, 20 pg/ml). However, *Il12a* mRNA levels were reduced in *Ifnar1*^−/−^ macrophages compared with WT at 6 h postinfection (Fig. 2C). Additionally, TNF-α protein production and *Tnfa* mRNA levels were also reduced in *Ifnar1*^−/−^ compared with WT macrophages following *M. tuberculosis* infection (Fig. 2C). In contrast to these cytokines, the secretion of IL-1β was increased in *Ifnar1*^−/−^ macrophages in response to *M. tuberculosis* infection as compared with WT cells, despite *Il1b* mRNA levels also being reduced at 6 h (Fig. 2D). Collectively, these results suggest that although high levels of IFN-β inhibit the ability of macrophages to make proinflammatory cytokines, paradoxically, autocrine type I IFN signaling is required in macrophages for optimal production of IL-12p40 and TNF-α following *M. tuberculosis* infection but still inhibits macrophage production of IL-1β.

### Timing of IFN signaling determines effects on macrophage cytokine production upon *M. tuberculosis* infection

**FIGURE 3.**
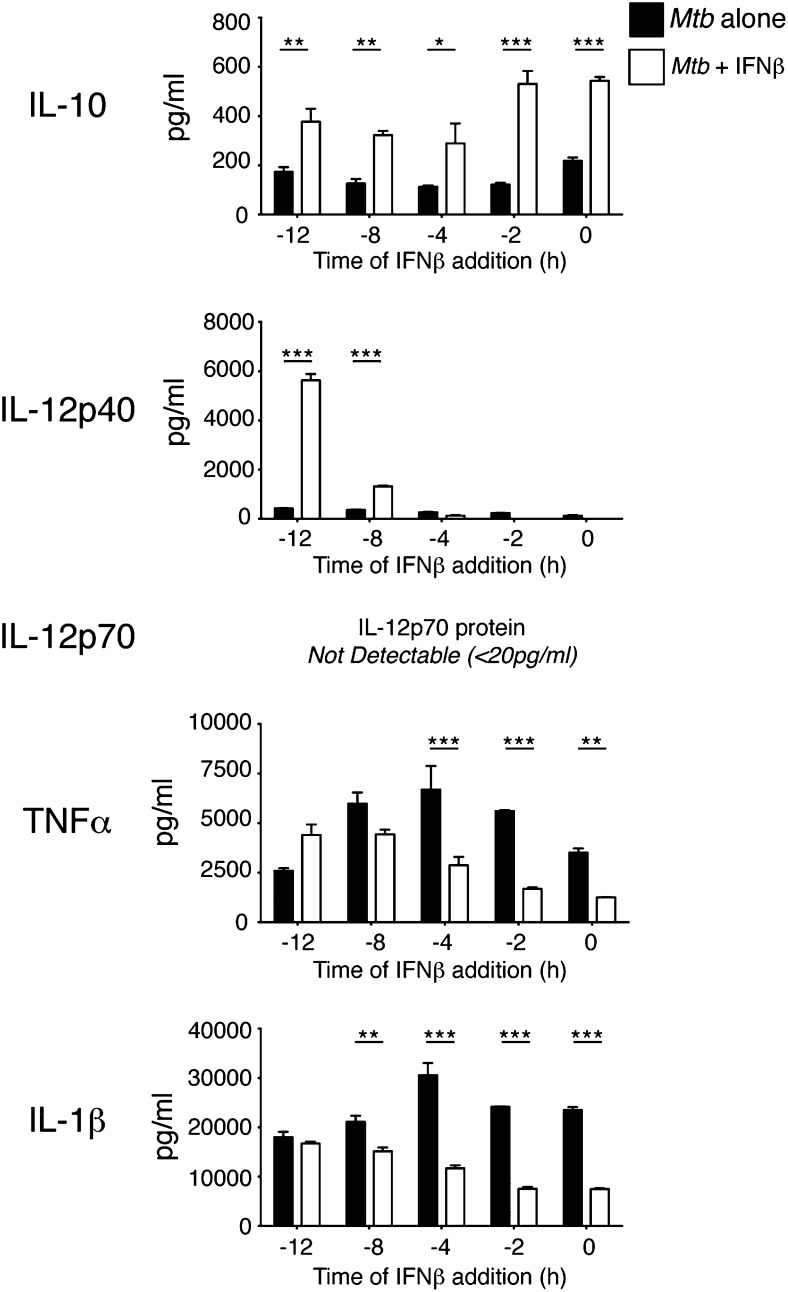
IFN-β pretreatment of *M. tuberculosis*–infected macrophages enhances IL-12 and TNF-α production. WT macrophages were infected with *M. tuberculosis* alone or in the presence of IFN-β (2 ng/ml), added at the indicated times prior to infection. Cytokine levels in culture supernatants were determined by ELISA at 24 h postinfection. Uninfected control samples were below the detection limit (20 pg/ml) for the cytokines measured (data not shown). Graphs show means ± SEM of triplicate samples. Data are representative of two independent experiments. Significance was determined using a two-way ANOVA with a Bonferroni post hoc test. *p < 0.05, **p < 0.01, ***p < 0.001.

To investigate whether timing of type I IFN signaling relative to *M. tuberculosis* infection might explain the differences in cytokine production observed between addition of recombinant type I IFN and autocrine type I IFN signaling, we pretreated macrophages for varying times prior to *M. tuberculosis* infection. Pretreatment of macrophages with IFN-β for 8 or 12 h prior to *M. tuberculosis* infection enhanced IL-12p40, and to a lesser extent TNF-α production, indicating that timing of IFN signaling relative to signaling through pattern recognition receptors is important in determining the effects of type I IFN in regulating the secretion of these proinflammatory cytokines (Fig. 3). IL-12p70 protein was not detected in any group (LOD, 20 pg/ml). IL-10 levels were enhanced by pre-addition of IFN-β at all time points tested but had the greatest effect when added at or shortly before *M. tuberculosis* infection (Fig. 3). IL-1β production by macrophages upon *M. tuberculosis* infection was inhibited by pre-addition of type I IFN, at most pretreatment time points investigated, although this did not significantly affect IL-1β levels at 12 h of pretreatment (Fig. 3). Again, the greatest effect was seen when IFN-β was added close to the time of infection.

### Exogenous IFN-β inhibits macrophage responsiveness to concomitant IFN-γ addition

**FIGURE 4.**
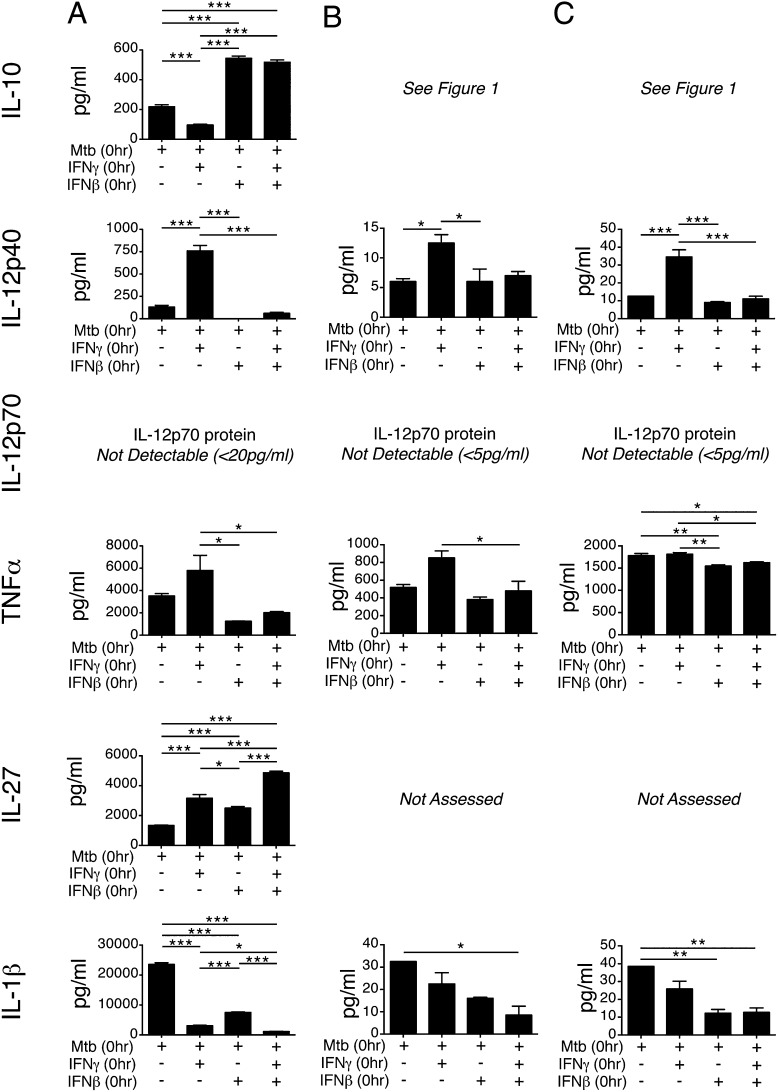
Exogenous IFN-β inhibits *M. tuberculosis*–infected macrophage responsiveness to concomitant IFN-γ addition. (**A**) WT macrophages were infected with *M. tuberculosis* alone or with *M. tuberculosis* and IFN-β (2 ng/ml), *M. tuberculosis* and IFN-γ (5 ng/ml), or *M. tuberculosis* and both IFN-β (2 ng/ml) and IFN-γ (5 ng/ml) together, added at the time of infection. Cytokine levels in culture supernatants were determined at 24 h postinfection. Uninfected control samples were below the detection limit (20 pg/ml) for the cytokines measured (data not shown). Graphs show means ± SEM. Significance was determined using a one-way ANOVA with a Bonferroni post hoc test. Data are representative of three independent experiments. (**B**) Myeloid cells (Lin^−^Ly6c^+^Ly6G^−^) were sorted from the lungs of WT mice and infected with *M. tuberculosis* and treated with IFN as in (A). Cytokine levels in culture supernatant were determined at 24 h postinfection using a Luminex bead array. Uninfected control samples were below the detection limit (5 pg/ml) for the cytokines measured (data not shown). Graphs show means ± SEM. Data are representative of two independent experiments. (**C**) Myeloid cells (Lin^−^Ly6c^+^Ly6G^−^) were sorted from the BM of WT mice and infected with *M. tuberculosis* and treated with IFN as in (A) and (B). Cytokine levels in culture supernatant were determined at 24 h postinfection using a Luminex bead array. Uninfected control samples were below the detection limit for the cytokines measured (data not shown). Graphs show means ± SEM. Data are representative of three independent experiments. *p < 0.05, **p < 0.01, ***p < 0.001.

IFN-γ is crucial to the host response against *M. tuberculosis* infection (10, 14) and is known to induce or enhance production of important host-protective cytokines such as IL-12 and TNF-α by macrophages and other myeloid cells while inhibiting IL-10 production (5, 9, 13, 14, 51–53). Because exogenous type I IFN and exogenous IFN-γ appear to have opposing effects on macrophages, we investigated which IFN type would have a dominant effect in influencing macrophage cytokine production following *M. tuberculosis* infection. To test this we infected WT macrophages with *M. tuberculosis*, also adding IFN-γ and IFN-β at the time of infection (Fig. 4). *M. tuberculosis*–infected macrophages treated singly with IFN-γ or IFN-β or left untreated were included as controls.

As expected, IL-10 production was inhibited in *M. tuberculosis*–infected macrophages treated with IFN-γ alone and enhanced in *M. tuberculosis*–infected macrophages treated with IFN-β alone (Fig. 4A). When both IFN-γ and IFN-β were added to macrophages infected with *M. tuberculosis*, IL-10 production was enhanced to a level at least equal to that seen with IFN-β alone, suggesting that IFN-β was overriding IFN-γ–mediated inhibition of IL-10 (Fig. 4A). *Il10* mRNA levels at 6 h postinfection were similarly affected by IFN treatment (Supplemental Fig. 2). A similar trend was seen with IL-12p40 production by *M. tuberculosis*–infected macrophages; that is, whereas IFN-γ alone greatly promoted IL-12p40 production, IFN-β alone and IFN-β together with IFN-γ resulted in markedly reduced levels of IL-12p40 (Fig. 4A). Similar results were found at the transcriptional level with *Il12a* and *Il12b* mRNA (Supplemental Fig. 2). TNF-α production by macrophages infected with *M. tuberculosis* was similarly affected, with IFN-γ alone promoting TNF-α, whereas IFN-β alone and IFN-β/IFN-γ together resulted in inhibition of TNF-α production (Fig. 4A). *Tnfa* mRNA, however, did not follow this trend, being downregulated by both IFN-γ and IFN-β (Supplemental Fig. 2). Effects on IL-27 and IL-1β were the exception to the trend of IFN-β overriding IFN-γ. IL-27 production was upregulated by both IFN-γ and IFN-β and further upregulated when both were added concomitantly (Fig. 4A). IFN-γ alone and IFN-β alone treatment both inhibited IL-1β production from *M. tuberculosis*–infected macrophages, although IFN-γ was slightly more potent than IFN-β (Fig. 4A). When both IFN-β and IFN-γ were added to *M. tuberculosis*–infected macrophages, IL-1β levels were reduced to a level below the single cytokine treatment groups (Fig. 4A). mRNA levels were similarly affected (Supplemental Fig. 2). In addition to adding both IFN to macrophages at the time of infection, we also repeated the experiment with 2 h of IFN-β pretreatment, prior to infection and IFN-γ addition, or 2 h of IFN-γ pretreatment, followed by infection and IFN-β addition, and we found the same results in both cases as adding both IFN at the time of infection that type I IFN effects are dominant (data not shown). Type I IFN has been shown to inhibit IFN-γ responsiveness during *L. monocytogenes* infection through inhibiting transcription of the gene encoding IFN-γ receptor subunit 1 (43, 54). We therefore examined mRNA levels of *Ifngr1* during *M. tuberculosis* infection of macrophages. Downregulation of *Ifngr1* mRNA levels upon *M. tuberculosis* infection could be observed, but this was only partially dependent on type I IFN signaling (data not shown), illustrating the complexity of this system.

Finally, to confirm that similar responses to IFN-β and IFN-γ were seen in ex vivo–derived cells, primary myeloid cells were sorted from the lungs and BM of WT mice, *M. tuberculosis* infected, and IFN treated as for experiments with BMDMs (Fig. 4B, 4C). Although overall levels of IL-12p40 were low in supernatant from *M. tuberculosis*–infected lung and BM myeloid cells, these cells responded similarly to BMDMs upon IFN treatment, with IFN-γ enhancing IL12p40 levels and IFN-β treatment inhibiting IFN-γ–mediated IL-12p40 production (Fig. 4B, 4C). IL-12p70 was not detected under any condition (Fig. 4). For TNF-α production, lung myeloid cells also showed a similar pattern of response to *M. tuberculosis* infection and IFN treatment as for IL-12p40, with the upregulation induced by IFN-γ being repressed by IFN-β treatment (Fig. 4B). In BM myeloid cells levels of TNF-α were not affected by IFN-γ compared with *M. tuberculosis* infection alone, whereas IFN-β treatment resulted in a modest, but significant reduction compared with *M. tuberculosis* infection alone and *M. tuberculosis* infection plus IFN-γ (Fig. 4C). Similar to findings in BMDMs, IL-1β production by *M. tuberculosis*–infected lung and BM myeloid cells was inhibited by both IFN-γ and IFN-β (Fig. 4B, 4C).

Collectively, these results suggest that exogenous type I IFN powerfully overcomes the macrophage response to IFN-γ during *M. tuberculosis* infection. However, this effect is not universal, as seen with IL-1β production, where both cytokines are inhibitory.

### Exogenous IFN-β inhibits IL-12, TNF-α, and IL-1β production in *M. tuberculosis*–infected macrophages through IL-10–dependent and –independent mechanisms

**FIGURE 5.**
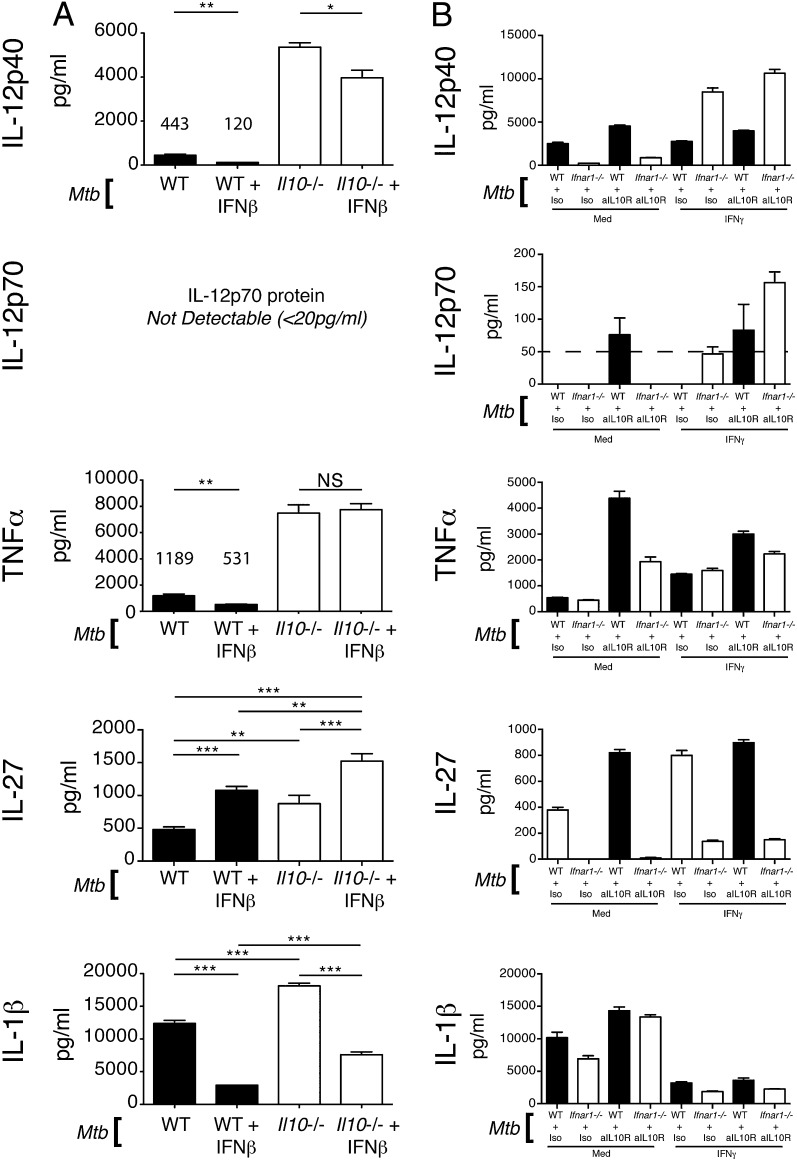
Exogenous IFN-β and endogenous type I IFN signaling affect proinflammatory cytokine production and responsiveness to IFN-γ in *M. tuberculosis*–infected macrophages through IL-10–dependent and –independent mechanisms. (**A**) WT and *Il10*^−/−^ macrophages were infected with *M. tuberculosis* or with *M. tuberculosis* and IFN-β (2 ng/ml), added at the time of infection. Levels of the indicated cytokines in culture supernatant were determined at 24 h postinfection by ELISA. Graphs show means ± SEM of triplicate samples. Significance was determined using a one-way ANOVA with a Bonferroni post hoc test. (**B**) WT and *Ifnar1*^−/−^ macrophages were infected with *M. tuberculosis*, treated with anti–IL-10R or isotype control Abs and with IFN-γ (5 ng/ml) or not, added at the time of infection. Levels of the indicated cytokines in culture supernatant were determined at 24 h postinfection by ELISA. Uninfected control samples were below the detection limit (20 pg/ml) for the cytokines measured (data not shown). Graphs show means ± SEM of triplicate samples. Data are representative of three independent experiments. *p < 0.05, **p < 0.01, ***p < 0.001.

IL-10 is a prominent inhibitor of myeloid cell functions (55). Given our previous results, we hypothesized that type I IFN was mediating its suppressive effects on proinflammatory cytokine production by *M. tuberculosis*–infected macrophages through induction of IL-10. To test this we infected IL-10–deficient macrophages with *M. tuberculosis* with and without addition of IFN-β (Fig. 5A). *Il10*^−/−^ macrophages produced greatly increased levels of IL-12p40 compared with WT cells upon *M. tuberculosis* infection (Fig. 5A). Addition of IFN-β to *Il10*^−/−^ macrophages reduced IL-12p40 levels, but not to the same extent as IFN-β added to WT macrophages infected with *M. tuberculosis* (∼25% reduction in *Il10*^−/−^ cells versus ∼70% in WT cells) (Fig. 5A). These results suggested that the inhibitory effect of IFN-β on IL-12p40 production is largely dependent on IL-10. IL-12p70 was not detectable in any group. TNF-α production was similarly increased in *Il10*^−/−^ *M. tuberculosis*–infected macrophages compared with WT cells (Fig. 5A). IFN-β treatment did not result in reduced TNF-α in *Il10*^−/−^ macrophages, indicating that the inhibitory effects of IFN-β on TNF-α production are totally dependent on IL-10 (Fig. 5A). Although IL-10 reduced the ability of macrophages to produce IL-27 following *M. tuberculosis* infection, it did not greatly affect the ability of IFN-β treatment to enhance IL-27 production (Fig. 5A). IL-1β production was only increased by 30% in *M. tuberculosis*–infected *Il10*^−/−^ macrophages compared with WT controls. IFN-β treatment was still able to significantly inhibit IL-1β production by IL-10–deficient macrophages infected with *M. tuberculosis* (Fig. 5A). This suggested that type I IFN had a dominant inhibitory effect on IL-1β that was minimally dependent on IL-10 during *M. tuberculosis* infection of macrophages.

### Type I IFN signaling impairs IFN-γ effects on *M. tuberculosis*–infected macrophage cytokine production via IL-10–dependent and –independent mechanisms

As described above, IFN-γ is crucial to the host response against *M. tuberculosis* infection (10, 14) and is known to induce or enhance production of important host-protective cytokines such as IL-12 (5, 9, 13, 14, 51–53). We noted that even in the absence of type I IFN or IL-10 signaling macrophages could not be induced to consistently make significant amounts of IL-12p70, the biologically active form of IL-12, upon *M. tuberculosis* infection. This indicated that removal of inhibitory signals alone might not be sufficient for induction of robust levels of this cytokine and that IFN-γ may be required.

To confirm that IFN-γ was required for IL-12p70 production in *M. tuberculosis*–infected macrophages and to test whether type I IFN together with IL-10 might exert negative regulatory effects on IFN-γ action, we infected WT or *Ifnar1*^−/−^ macrophages with *M. tuberculosis* in the presence or absence of rIFN-γ and anti–IL-10R or anti–IL-10 Abs (Fig. 5B and data not shown). IL-12p40 production could be enhanced in *M. tuberculosis*–infected WT macrophages either treated with IFN-γ or anti–IL-10R and was further enhanced in *M. tuberculosis*–infected *Ifnar1*^−/−^ macrophages treated with IFN-γ or IFN-γ and anti–IL-10R (Fig. 5B). Addition of IFN-γ, a known inducer of IL-12p70 in concert with TLR signals (51, 53, 56), induced little to no IL-12p70 in *M. tuberculosis*–infected WT macrophages (Fig. 5B). However, in the absence of type I IFN signaling or upon Ab blockade of the IL-10R, IFN-γ was able to induce robustly detectable levels of IL-12p70 production (Fig. 5B). TNF-α production by *M. tuberculosis*–infected macrophages was also enhanced upon IFN-γ treatment, and this was not further increased by abrogation of the type I IFN receptor but was increased upon anti–IL-10R blockade (Fig. 5B). As expected, IL-27 levels were greatly impaired in *Ifnar1*^−/−^ BMDMs compared with WT, and although anti–IL-10R blockade and IFN-γ treatment increased IL-27 in both WT and *Ifnar1*^−/−^, levels in the type I IFN receptor–deficient BMDMs could not be rescued to those of the WT controls (Fig. 5B).

IL-1β production by both WT and *Ifnar1*^−/−^ *M. tuberculosis*–infected macrophages was greatly inhibited by IFN-γ addition (Fig. 5B). The relative level of inhibition was similar between WT and *Ifnar1*^−/−^ macrophages, although there was a trend to greater inhibition in the *Ifnar1*^−/−^ macrophages (Fig. 5B).

Altogether, these results are in agreement with previous studies reporting that IFN-γ is an important inducer and/or enhancer of IL-12 and TNF-α production. However, we now show that during *M. tuberculosis* infection of macrophages the ability of IFN-γ to positively regulate production of these cytokines is significantly impaired by both autocrine type I IFN and IL-10. Production of bioactive IL-12p70, in particular, is strongly suppressed by these two negative regulatory factors.

### Type I IFN regulation of IL-1β production is dependent on inducible NO synthase and IL-10

**FIGURE 6.**
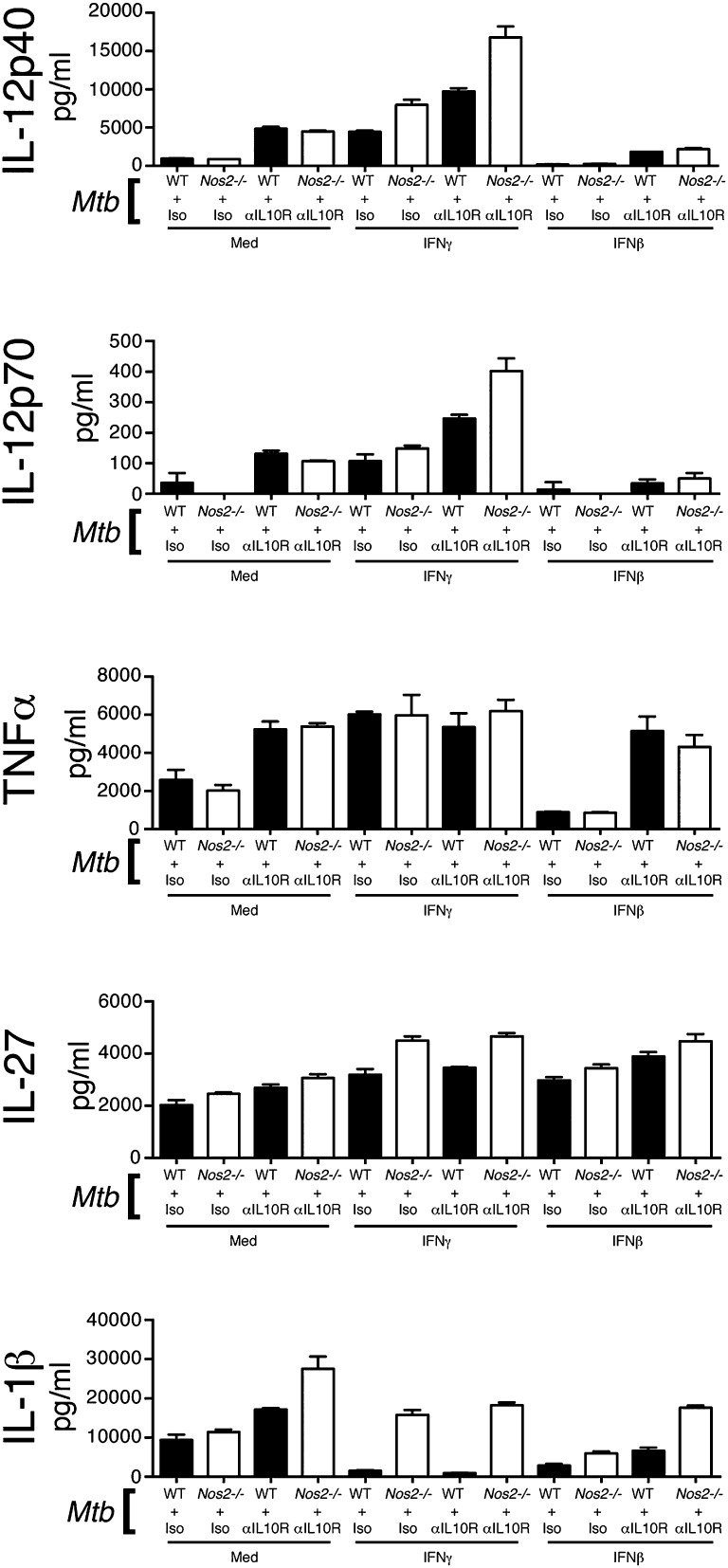
Type I IFN regulation of IL-1β production is dependent on iNOS and IL-10. WT and *Nos2*^−/−^ macrophages were infected with *M. tuberculosis*, treated with anti–IL-10R or isotype control Abs and with either IFN-γ (5 ng/ml) or IFN-β (2 ng/ml) or media, added at the time of infection. Levels of the indicated cytokines in culture supernatant were determined at 24 h postinfection by ELISA. Uninfected control samples were below the detection limit (20 pg/ml) for the cytokines measured (data not shown). Graphs show means ± SEM of triplicate samples. Data are representative of at least three independent experiments.

In contrast to type I IFN effects on TNF-α, we found that inhibition of IL-1β (and IL-12) production by IFN-β was only partially dependent on IL-10. Additionally, we observed co-operative inhibition of IL-1β by IFN-γ and IFN-β, in contrast to their usually cross-regulatory effects on each other’s action on cytokine production. IFN-γ has recently been described to negatively regulate IL-1β production during *M. tuberculosis* infection in an inducible NO synthase (iNOS, gene name *Nos2*)–dependent manner (57). Because type I IFN can also induce iNOS, we hypothesized that IFN-β was also inhibiting IL-1β production through upregulation of iNOS. To test this we infected WT and *Nos2*^−/−^ BMDMs with *M. tuberculosis* and concomitantly added IFN-γ or IFN-β (Fig. 6). We also included groups treated with anti–IL-10R blocking Abs to compare IL-10 effects versus iNOS effects (Fig. 6).

In macrophages infected with *M. tuberculosis* alone, abrogation of *Nos2* had only minor effects on IL-1β production (Fig. 6). However, in *M. tuberculosis*–infected *Nos2*^−/−^ BMDMs treated with anti–IL-10R Abs levels of IL-1β were elevated over levels in infected WT, anti–IL-10R-treated BMDMs, suggestive of IL-10 regulation of iNOS and/or its function (Fig. 6). When *M. tuberculosis*–infected WT macrophages were treated with IFN-γ, IL-1β production was almost entirely abrogated and this abrogation was completely relieved in infected *Nos2*^−/−^ BMDMs treated with IFN-γ (Fig. 6). This suggests that iNOS mediates suppression of IL-1β production by IFN-γ, in agreement with the findings of Mishra et al. (57). IL-10R blockade had little effect on macrophages treated with IFN-γ, likely due to IFN-γ suppressing IL-10 production (Fig. 6; see Fig. 4). As expected, IL-1β production by *M. tuberculosis*–infected macrophages was also suppressed by treatment with IFN-β (Fig. 6). This was partially relieved by the abrogation of iNOS, but not to the same extent as seen in IFN-γ–treated groups (Fig. 6). Ab blockade of IL-10R in IFN-β–treated, *M. tuberculosis*–infected macrophages lacking *Nos2* did restore IL-1β levels to those of WT infected, untreated macrophages, suggesting that IFN-β inhibits IL-1β production through the dual mechanisms of IL-10 and iNOS (Fig. 6).

Interestingly, we also noted that although iNOS did not greatly affect production of the other cytokines measured in *M. tuberculosis*–infected alone macrophages or *M. tuberculosis*–infected and IFN-β–treated macrophages, in macrophages infected with *M. tuberculosis* and treated with IFN-γ, levels of IL-12 and IL-27 were elevated in the absence of *Nos2* (Fig. 6). This suggests that iNOS also acts as a feedback negative regulator of IFN-γ function.

### Type I IFN signaling inhibits macrophage restriction of *M. tuberculosis* growth and killing in response to IFN-γ

**FIGURE 7.**
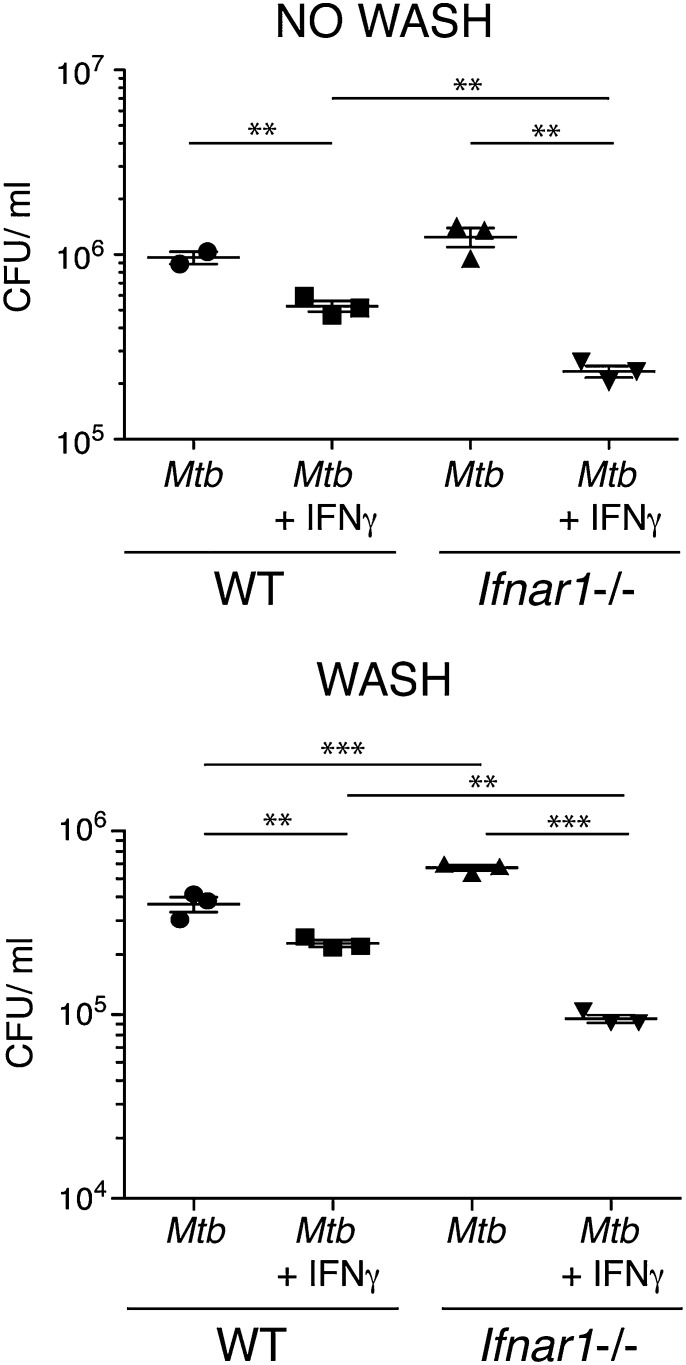
Type I IFN signaling inhibits macrophage restriction of *M. tuberculosis* growth and killing in response to IFN-γ. WT and *Ifnar1*^−/−^ macrophages were infected with *M. tuberculosis* in the presence or absence of IFN-γ (5 ng/ml), added at the time of infection. At 4 h postinfection media containing *M. tuberculosis* was removed, cells were washed in PBS, and fresh media (with no *M. tuberculosis*) were replaced (*bottom panel*) or not (*top panel*). At 96 h postinfection cells were washed in PBS, lysed in 0.2% saponin, and bacterial loads were enumerated via serial dilution and plating. Line and bars show means ± SEM. Significance was determined using an unpaired *t* test. Data are representative of at least two independent experiments for each of the two systems (i.e., wash or no wash). **p < 0.01, ***p < 0.001.

In addition to induction of crucial host-protective cytokines, IFN-γ is known to be important in activating macrophages to restrict intracellular bacterial growth, including that of *M. tuberculosis* (5, 6, 11, 12, 58, 59). Indeed, IFN-γ has been shown to be the crucial cytokine for induction of the reactive nitrogen and oxygen species that are necessary for antimicrobial activity in murine macrophages (60). In view of our previous results showing that IFN-β interferes with the IFN-γ–mediated regulation of several cytokines, we next investigated whether type I IFN signaling could also affect IFN-γ–mediated restriction of *M. tuberculosis* in infected macrophages. To study this, WT and *Ifnar1*^−/−^ macrophages were infected with *M. tuberculosis* in the presence or absence of IFN-γ, and at 96 h postinfection bacterial loads were assessed (Fig. 7). We employed two methods of infection for these experiments: either leaving bacteria in the well for the full 96 h postinfection (Fig. 7, *top*) or washing out after 4 h of infection (Fig. 7, *bottom*), with matching results being observed between the two. Additionally, we controlled for differences in bacterial uptake between WT and *Ifnar1*^−/−^ macrophages by enumerating CFUs at 4 h postinfection, and we found no difference between the two cell types (data not shown). Bacterial loads were not significantly different between WT and *Ifnar1*^−/−^ macrophages infected with *M. tuberculosis* alone with no wash but were slightly increased in *Ifnar1*^−/−^ cells versus WT when the *M. tuberculosis* inoculum was washed out (Fig. 7). IFN-γ treatment induced a reduction in bacterial load in WT macrophages of ∼2-fold compared with *M. tuberculosis* infection alone, but treatment of *Ifnar1*^−/−^ macrophages with IFN-γ led to a reduction in bacterial load of >5-fold compared with *Ifnar1*^−/−^ macrophages infected with *M. tuberculosis* alone (Fig. 7). Addition of IFN-β to *M. tuberculosis*–infected WT macrophages did not alter bacterial loads compared with *M. tuberculosis* infection alone (data not shown). Treatment of *M. tuberculosis*–infected macrophages with IFN-γ and IFN-β together did not alter bacterial loads compared with *M. tuberculosis*–infected macrophages treated with IFN-γ alone (data not shown). These results suggest that endogenous levels of type I IFN induced by *M. tuberculosis* infection can inhibit the ability of IFN-γ to restrict bacterial levels in *M. tuberculosis*–infected macrophages, in addition to inhibiting IFN-γ effects on macrophage cytokine production during *M. tuberculosis* infection.

## Discussion

There is growing evidence in both mice and humans that type I IFNs are detrimental to host resistance during TB (15, 16, 18–25, 29). However, the mechanisms by which type I IFN negatively impacts the host response to *M. tuberculosis* are not well understood. Several recent studies have highlighted the importance of type I IFN in the myeloid cell response to *M. tuberculosis* (16, 20, 21, 25). In this study, we show that type I IFN signaling is required for macrophage induction of the immunosuppressive cytokine IL-10 during *M. tuberculosis* infection. Type I IFN is also responsible for suppressing production of several proinflammatory cytokines by macrophages, particularly IL-12, TNF-α, and IL-1β, which are considered crucial to host protection against *M. tuberculosis* (7, 8, 20, 61–64). IL-10 is an important mediator of the suppressive effect on IL-12 and TNF-α production. IFN-γ is a key cytokine for activation of myeloid cells to both produce increased levels of protective cytokines, particularly IL-12, and to restrict intracellular bacterial growth (5, 6, 9, 11–13, 58, 65). Importantly, we now show that type I IFN dramatically suppresses the macrophage response to IFN-γ for induction of IL-12p40 and IL-12p70, as well as for *M. tuberculosis* growth restriction and killing.

We first investigated the effect of type I IFN on IL-10 production by macrophages infected with *M. tuberculosis* and found both a requirement for type I IFN signaling in induction of IL-10 and an increase in IL-10 production following addition of exogenous IFN-β to *M. tuberculosis*–infected macrophages, in agreement with several other studies in different systems, such as LPS stimulation (30–32). Analysis of *Il10* mRNA levels suggested that type I IFN mediated its effects on IL-10 production through regulating initial transcription of the *Il10* gene but also through stabilization of *Il10* transcripts. IL-27 has previously been reported as a mediator of IL-10 induction downstream of type I IFN following LPS stimulation of BMDMs (32). This induction of IL-10 by IL-27 was reported to be dependent on STAT-1 and STAT-3 activation (32). We found that type I IFN was required for induction of IL-27 in *M. tuberculosis*–infected macrophages and that addition of IFN-β enhanced IL-27 production, in agreement with this study and others (32, 66). However, we found no requirement for IL-27 signaling in IL-10 induction or enhancement of IL-10 production by IFN-β. IL-10 production by BM-derived dendritic cells and BM monocytes was also unaffected by IL-27. Furthermore, our BMDMs did not activate STAT-1 and STAT-3 following IL-27 treatment, in keeping with another study (48) that also did not detect STAT-1 and STAT-3 phosphorylation in BMDMs following IL-27 treatment. In this study the authors could not detect induction of STAT-1–dependent genes in BMDMs following IL-27 treatment and reported low levels of IL-27R expression, suggesting that these cells were “minimally responsive” to IL-27 (48). We also found low relative levels of IL-27R expression in our BMDMs, in accordance with this study (48) and others who found low relative levels of IL-27R expression on F4/80^+^ splenic macrophages (67) and plastic adherent splenic macrophages (50), whereas we show that T cells do express the IL-27R, as others report (50, 67). Although this lack of STAT-1 and STAT-3 activation is in contrast to Iyer et al. (32), the reasons for the difference between our findings and those of Iyer et al. are presently unclear, but may be due to laboratory differences in generation and culture conditions of BMDMs. Note that our findings do not exclude IL-27 responsiveness by other types of macrophage or myeloid cell that may also respond in a manner not assessed in this study (68, 69).

We also investigated the effects of type I IFN on proinflammatory cytokine production. Addition of IFN-β to *M. tuberculosis*–infected macrophages potently suppressed IL-12p40 production, in agreement with a number of studies in viral and some bacterial models showing suppression of IL-12 by type I IFN (24, 70–73). TNF-α and IL-1β production were also suppressed by type I IFN addition. Interestingly, and in contrast to these findings, autocrine type I IFN was also partially required for optimal IL-12p40 and TNF-α production by macrophages following *M. tuberculosis* infection, in agreement with a previous study where type I IFN signaling was required for IL-12 production following TLR stimulation (74). The discrepancy between our results with addition of IFN-β versus deficiency in type I IFN signaling appears to be due to the timing of type I IFN signaling relative to signaling through pattern recognition receptors, because 12 h of pretreatment with IFN-β resulted in increased levels of IL-12 and TNF-α upon *M. tuberculosis* infection, whereas IFN-β treatment proximal to *M. tuberculosis* infection consistently resulted in suppression of IL-12p40 and TNF-α production. This may indicate that distinct mechanistic programs of action of type I IFN exist, dependent on proximity to other stimuli (such as infection).

In contrast to IL-12p40 and TNF-α, type I IFN signaling inhibited macrophage production of IL-1β protein following *M. tuberculosis* infection under all conditions tested. This is in clear agreement with recent studies in both mouse models (20) and human cells (21) and illustrates an important mechanism of type I IFN negative regulation of the host response, because IL-1 is crucial to protection during TB (20, 62–64).

Given that type I IFN induced IL-10 in *M. tuberculosis*–infected macrophages, we assessed whether IL-10 was mediating the inhibitory effects of type I IFN on IL-12p40, TNF-α, and IL-1β. IFN-β treatment of IL-10–deficient macrophages infected with *M. tuberculosis* revealed that IL-10 did indeed mediate much of the suppressive action of type I IFN on IL-12p40 production and was totally responsible for inhibition of TNF-α production. Low levels of TNF-α and IL-12 in the lungs of *M. tuberculosis*–infected mice have previously been associated with high type I IFN levels, although a role for IL-10 was not formally investigated (18). However, IL-10 appears to limit IL-12 production during mycobacterial infection (38) and also limits the Th1 response and associated IFN-γ production during *M. tuberculosis* infection in mice (39). These results suggest that IL-10 may be an important intermediary in type I IFN–mediated disruption of the Th1 response during *M. tuberculosis* infection through suppression of macrophage production of cytokines such as IL-12. IL-10 also partially mediated suppression of IL-1β production by *M. tuberculosis*–infected macrophages, in agreement with a recent study (20), but suggests that most of inhibition of IL-1β production by type I IFN in *M. tuberculosis*–infected macrophages is through IL-10–independent mechanisms. Analysis of *Nos2*-deficient macrophages suggested that iNOS is a significant mechanism additional to IL-10 through which type I IFN mediates its suppressive effects on IL-1β synthesis. Interestingly, in the context of IFN-γ treatment, iNOS was also found to negatively regulate production of other cytokines, including IL-12 and IL-27.

We noted that even in the absence of the negative regulation of type I IFN or IL-10, *M. tuberculosis*–infected macrophages rarely produced robustly detectable levels of the biologically active form of IL-12, IL-12p70. This suggested that IL-12p70 is under tight regulation by other factors and/or might require additional promoting signals for its induction in *M. tuberculosis*–infected macrophages. IFN-γ is known as an inducer of IL-12p70 (51, 53, 56) and as a crucial activator of macrophages during *M. tuberculosis* infection (5, 11, 12, 14). We therefore assessed the effects of IFN-γ stimulation on *M. tuberculosis*–infected macrophages and whether type I IFN (either autocrine or exogenous) could perturb macrophage responsiveness to IFN-γ. IFN-γ treatment enhanced IL-12p40 and TNF-α production while suppressing IL-10 and IL-1β in *M. tuberculosis*–infected macrophages. However, little to no IL-12p70 was induced by IFN-γ in WT macrophages infected with *M. tuberculosis*. Only in the absence of either type I IFN signaling or IL-10 could IFN-γ addition induce robustly detectable amounts of IL-12p70. This indicates that these two pathways act as important regulators of the macrophage response to IFN-γ during *M. tuberculosis* infection. In particular, the ability of IFN-γ to drive macrophage production of biologically active IL-12p70 in response to *M. tuberculosis* remains compromised when the type I IFN or IL-10 pathways are intact.

To mimic a situation in which an *M. tuberculosis*–infected macrophage responds to both type I IFN and IFN-γ present in the microenvironment, we infected macrophages with *M. tuberculosis* and concomitantly added type I IFN and IFN-γ. Strikingly, type I IFN completely abrogated the ability of IFN-γ to downregulate IL-10 production and enhance IL-12p40 and TNF-α production. Type I IFN therefore seems to play a dominant regulatory role in *M. tuberculosis*–infected macrophages, which IFN-γ is unable to overcome. How type I IFN blocks macrophage responsiveness to IFN-γ remains unclear. Type I IFN can downregulate IFN-γ receptor expression (43, 54) and inhibit signaling (75) to limit macrophage activation. In our system this mechanism is likely to be only partially active, because type I IFN did not block all effects of IFN-γ on macrophages and only partially mediated effects on *Ifngr1* transcript levels. In particular, the ability of IFN-γ to suppress IL-1β production was largely unaffected by IFN-β treatment, and if anything was actually increased by the presence of IFN-β. This suggests that the effects of type I IFN on macrophage IFN-γ responsiveness are specific, rather than global.

Myeloid cells such as macrophages are known to be primary reservoirs of *M. tuberculosis* bacteria during TB (1–3). A critical role of IFN-γ is therefore to activate these cells to restrict intracellular bacterial growth through induction of antimicrobial effectors such as iNOS (5, 6, 12, 14). Our data suggest that type I IFN signaling has inhibitory effects on IFN-γ–induced macrophage restriction of *M. tuberculosis*. The mechanism for this remains unclear but may involve inhibition of reactive oxygen species induction (76).

In summary, we show in the present study that type I IFN is a powerful negative regulator of the immune response to *M. tuberculosis* at the level of the infected macrophage. Type I IFN suppresses both initial proinflammatory cytokine production by infected macrophages, in large part through induction of high levels of IL-10, and also strongly inhibits the macrophage’s ability to respond to IFN-γ stimulation. Type I IFN may therefore act to interrupt the Th1 immune response, which is crucial to host resistance to *M. tuberculosis*, through suppressing production of the proinflammatory cytokines that prime this response but also through making macrophages unresponsive to subsequent IFN-γ feedback from Th1 cells and other IFN-γ sources. Additionally, type I IFN may act directly to prevent IFN-γ–mediated *M. tuberculosis* growth restriction by macrophages, allowing a niche of *M. tuberculosis* permissive cells to develop.

## Supplementary Material

Data Supplement
