# Supplementary material for: Type I IFN Induces IL-10 Production in an IL-27–Independent Manner and Blocks Responsiveness to IFN-γ for Production of IL-12 and Bacterial Killing in Mycobacterium tuberculosis–Infected Macrophages
Source: J Immunol. 2014 Sep 3;193(7):3600–12. doi: 10.4049/jimmunol.1401088 (PMC4170673; doi:10.4049/jimmunol.1401088)
Supplement: Data Supplement [file supp_193_7_3600__index.html]

Type I IFN Induces IL-10 Production in an IL-27–Independent Manner and Blocks Responsiveness to IFN-γ for Production of IL-12 and Bacterial Killing in Mycobacterium tuberculosis–Infected Macrophages — Data Supplement 

# Type I IFN Induces IL-10 Production in an IL-27–Independent Manner and Blocks Responsiveness to IFN-γ for Production of IL-12 and Bacterial Killing in *Mycobacterium tuberculosis*–Infected Macrophages

## Data Supplement

**Files in this Data Supplement:**

- Supplemental Figures 1 (PDF)
