# Supplementary material for: Type I IFN Induces IL-10 Production in an IL-27–Independent Manner and Blocks Responsiveness to IFN-γ for Production of IL-12 and Bacterial Killing in Mycobacterium tuberculosis–Infected Macrophages
Source: J Immunol. 2014 Sep 3;193(7):3600–12. doi: 10.4049/jimmunol.1401088 (PMC4170673; doi:10.4049/jimmunol.1401088)
Supplement: Data Supplement [file 1401088_JI_1401088_Supplemental_Figures_1.pdf]

Supplementary Figure 1

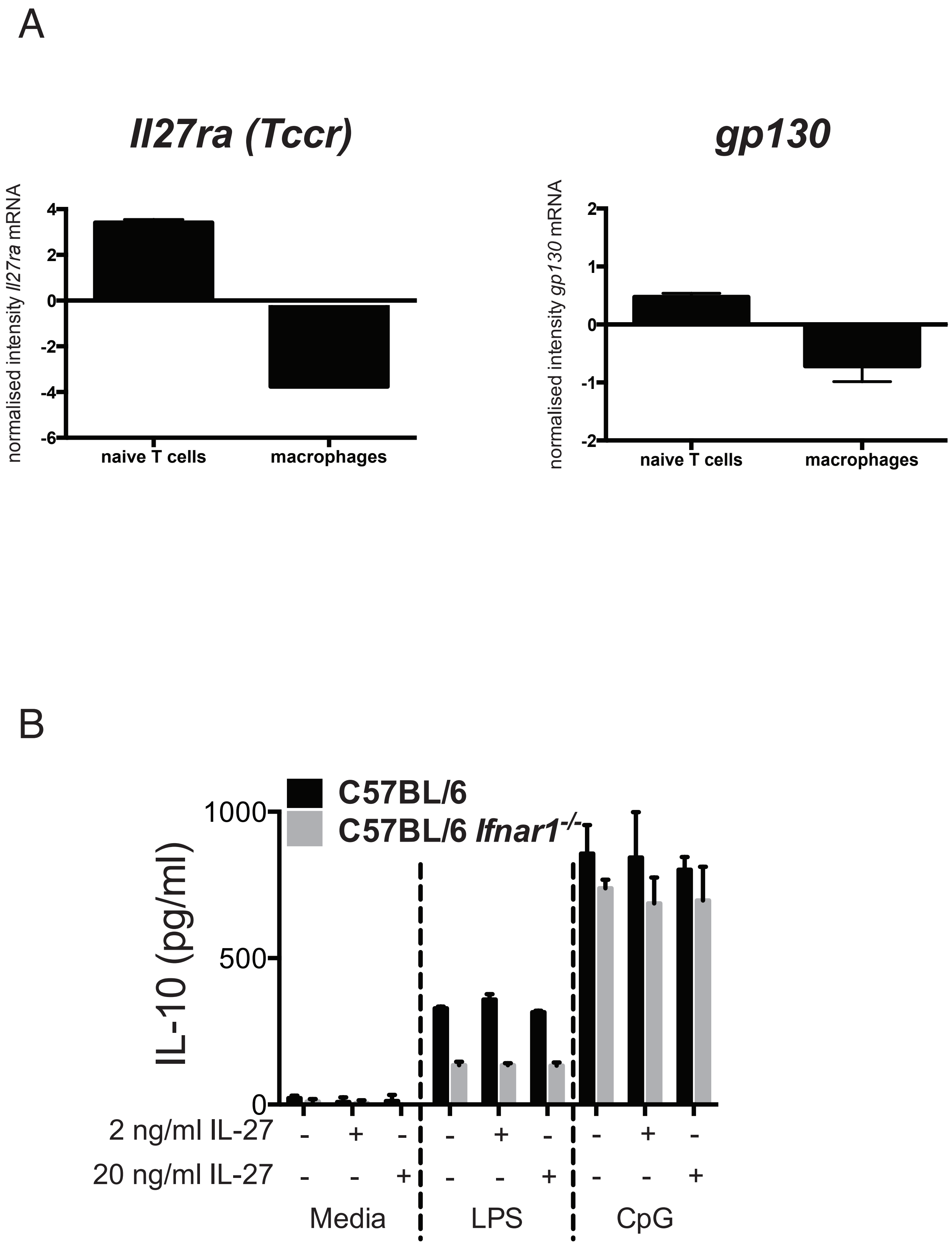

Supplementary Figure 1. Macrophage IL-27 receptor subunit mRNA expression relative to naïve T cells and IL-10 production in response to recombinant IL-27 treatment upon LPS or CpG treatment.

(A) mRNA levels of the IL-27 receptor subunits IL-27ra and gp130 in resting bone marrow-derived macrophages and naïve T cells were filtered by detection from background and levels normalised to the median of all samples. (B) WT and IFNAR-/- macrophages were stimulated with 10ng/ml LPS or 0.5 μM CpG and treated at the same time with recombinant IL-27 at the indicated doses. IL-10 levels in the supernatant were then measured by ELISA at 24 hours post-stimulation. Graphs show mean +/- SEM. Data is representative of four independent experiments.

Supplementary Figure 2

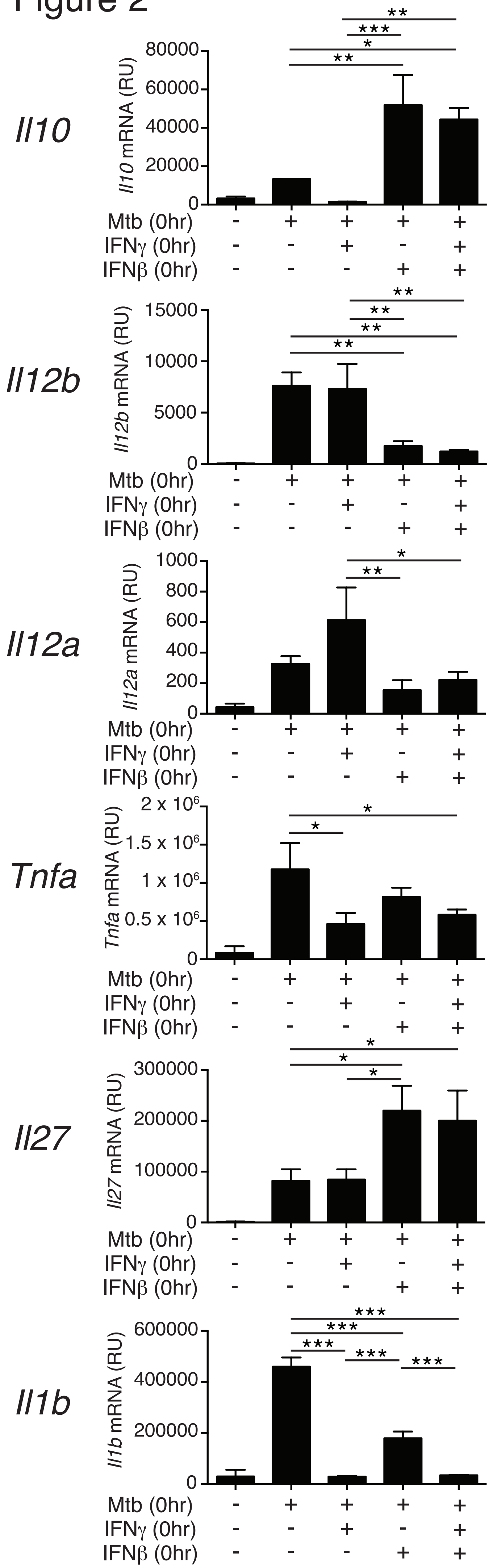

Supplementary Figure 2. Exogenous IFNβ inhibits *Mtb* infected macrophage responsiveness to concomitant IFNγ addition.

WT macrophages were infected with *Mtb* alone or with *Mtb* and IFNβ (2 ng/ml), *Mtb* and IFNγ (5 ng/ml) or *Mtb* and both IFNβ (2 ng/ml) and IFNγ (5 ng/ml) together, added at the time of infection. Cytokine mRNA levels were then measured by qRT-PCR at 6 h post-infection. Graphs show mean +/- SEM. Significance was determined using 1-way ANOVA with Bonferroni post-hoc test. Data is representative of three independent experiments.
